# Population genomics provides insights into the population structure and temperature-driven adaptation of *Collichthys lucidus*

**DOI:** 10.1186/s12864-021-08045-8

**Published:** 2021-10-08

**Authors:** Linlin Zhao, Fangyuan Qu, Na Song, Zhiqiang Han, Tianxiang Gao, Zhaohui Zhang

**Affiliations:** 1 First Institute of Oceanography, Ministry of Natural Resources, Qingdao, Shandong 266100 P. R. China; 2 Fisheries College, Ocean University of China, Qingdao, Shandong 266003 P. R. China; 3 School of Fishery, Zhejiang Ocean University, Zhoushan, Zhejiang 316004 P. R. China

**Keywords:** *Collichthys lucidus*, Genetic diversity, Population structure, Local adaptive, Population genomics

## Abstract

**Background:**

Understanding the genetic structure and local adaptive evolutionary mechanisms of marine organisms is crucial for the management of biological resources. As the ecologically and commercially important small-sized shallow-sea fish, *Collichthys lucidus* plays a vital role in the structure and functioning of marine ecosystem processes. *C. lucidus* has been shown to have an obvious population structure. Therefore, it is an ideal candidate for investigating population differentiation and local adaptation under heterogeneous environmental pressure.

**Results:**

A total of 184,708 high-quality single nucleotide polymorphisms (SNPs) were identified and applied to elucidate the fine-scale genetic structure and local thermal adaptation of 8 *C. lucidus* populations. Population structure analysis based on all SNPs indicated that the northern group and southern group of *C. lucidus* have a strong differentiation. Moreover, 314 SNPs were found to be significantly associated with temperature variation, and annotations of genes containing temperature-related SNPs suggested that genes were involved in material (protein, lipid, and carbohydrate) metabolism and immune responses.

**Conclusion:**

The high genetic differentiation of 8 *C. lucidus* populations may have been caused by long-term geographic isolation during the glacial period. Moreover, we suspected that variation in these genes associated with material (protein, lipid, and carbohydrate) metabolism and immune responses was critical for adaptation to spatially heterogeneous temperatures in natural *C. lucidus* populations. In conclusion, this study could help us determine how *C. lucidus* populations will respond to future ocean temperature rising.

**Supplementary Information:**

The online version contains supplementary material available at 10.1186/s12864-021-08045-8.

## Background

Inferring the genetic diversity, population structure and changing patterns of marine species is critical to the successful management of exploited populations, allowing conservation units to be identified and individuals to be assigned to geographical areas [1, 2]. However, assessing the current genetic structure and population connectivity of marine species remains a major challenge [3] because marine species usually have large population sizes and pelagic eggs. Moreover, the degree of connectivity between populations is often high due to less obvious geographic barriers in the oceans [4]. These biological characteristics may lead to high levels of internal genetic diversity and low levels of genetic differentiation between populations, even in marine species with a large distribution range [5]. Additionally, many marine species may have had insufficient time for divergence since the colonization of postglacial habitats [6]. In conclusion, it may be difficult to evaluate the genetic diversity and population structure of marine species, which may affect the reasonable management of these species.

Population genetics has a great advantage in correctly explaining the population genetic structure of marine species and exploring its influencing factors. Although previous studies have detected genetic differentiation patterns of marine species at a small spatial scale based on a small number of genetic markers [7], the deficiency of hierarchical analysis of variance based on F-statistics (*F*_ST_) is extremely obvious when it is applied to the genetics of large populations (even for species with weak migration abilities), which is especially obvious when single or limited genetic markers were used [8]. Therefore, it is essential to increase the number of genetic markers to help us understand the population structure of marine species in detail.

**Fig. 1:**
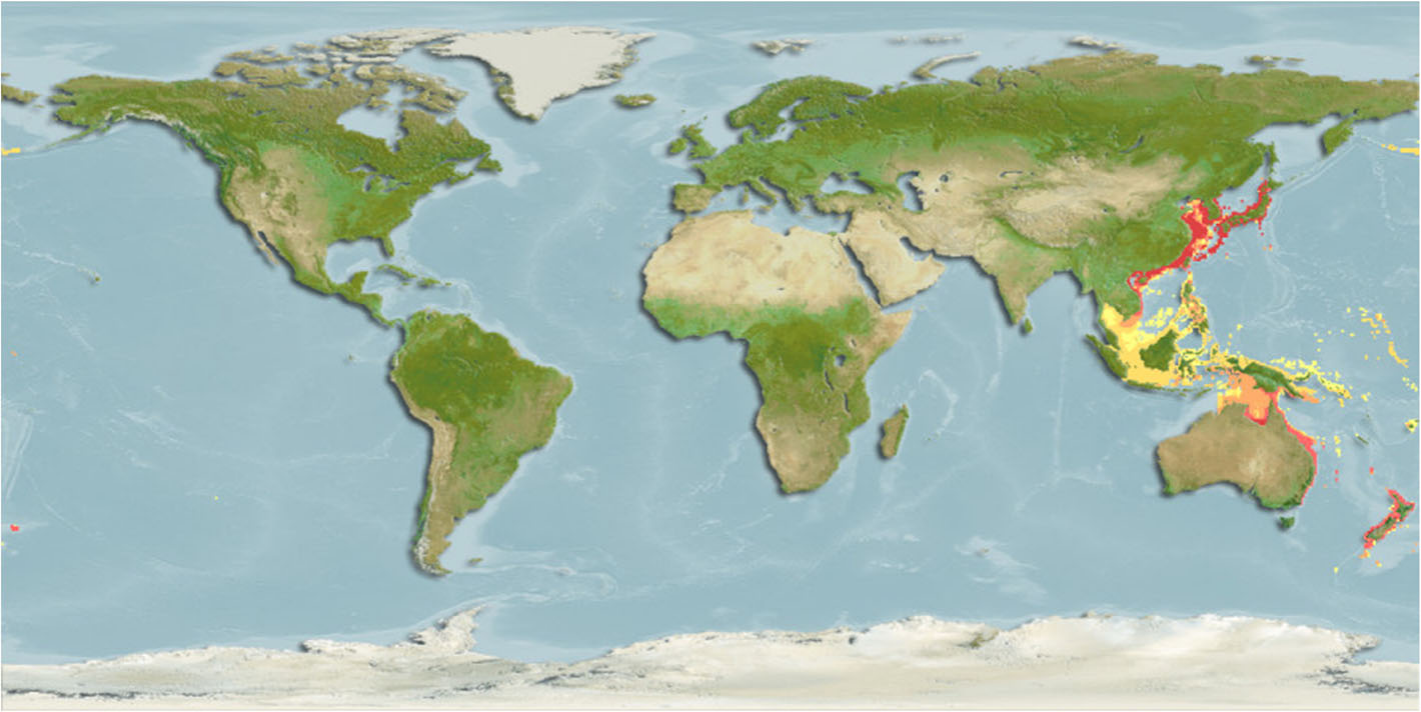
The distribution range of *C. lucidus* (https://www.fishbase.se/search)

*Collichthys lucidus* is a short-migratory shallow-sea fish that prefers brackish water in estuaries and has a life cycle including pelagic eggs [9, 10]. With rapid growth, high yield and rich nutrition, *C. lucidus* has become an important fresh seafood for residents in coastal areas. This species lives in warm water with low salinity and eats shrimp and crabs. As an ecologically and commercially important small-sized fish, *C. lucidus* is distributed along the inshore waters of China (Fig. 1) and also plays a vital role in the structure and functioning of marine ecosystem processes. Previous population studies based on mitochondrial DNA segments suggested that changes in the sea level during the Pleistocene limited the spread of *C. lucidus* and promoted the emergence of isolated populations, which ultimately had a significant impact on the systematic geographic pattern of this species [9, 11]. However, the accuracy of population structure research on *C. lucidus* is limited by the number of genetic markers used, because the hierarchical variance analysis algorithm is not suitable for population genetic studies with a limited number of genetic markers, even for populations with relatively weak migration abilities. Moreover, previous studies restricted to neutral genetic markers have provided limited insights into the local adaptation mechanisms of *C. lucidus*. Population genomics provides powerful genome-wide genotyping methods and holds great promise for population genetic studies, as it can allow the detection of local adaptation under temperature-driven pressure [12]. This is the case because population genomics may increase the power and resolution of traditional genetic approaches by increasing the number of variable genome-wide genetic markers. Population genomics can also reveal genetic variation in adaptive traits [13].

**Fig. 2:**
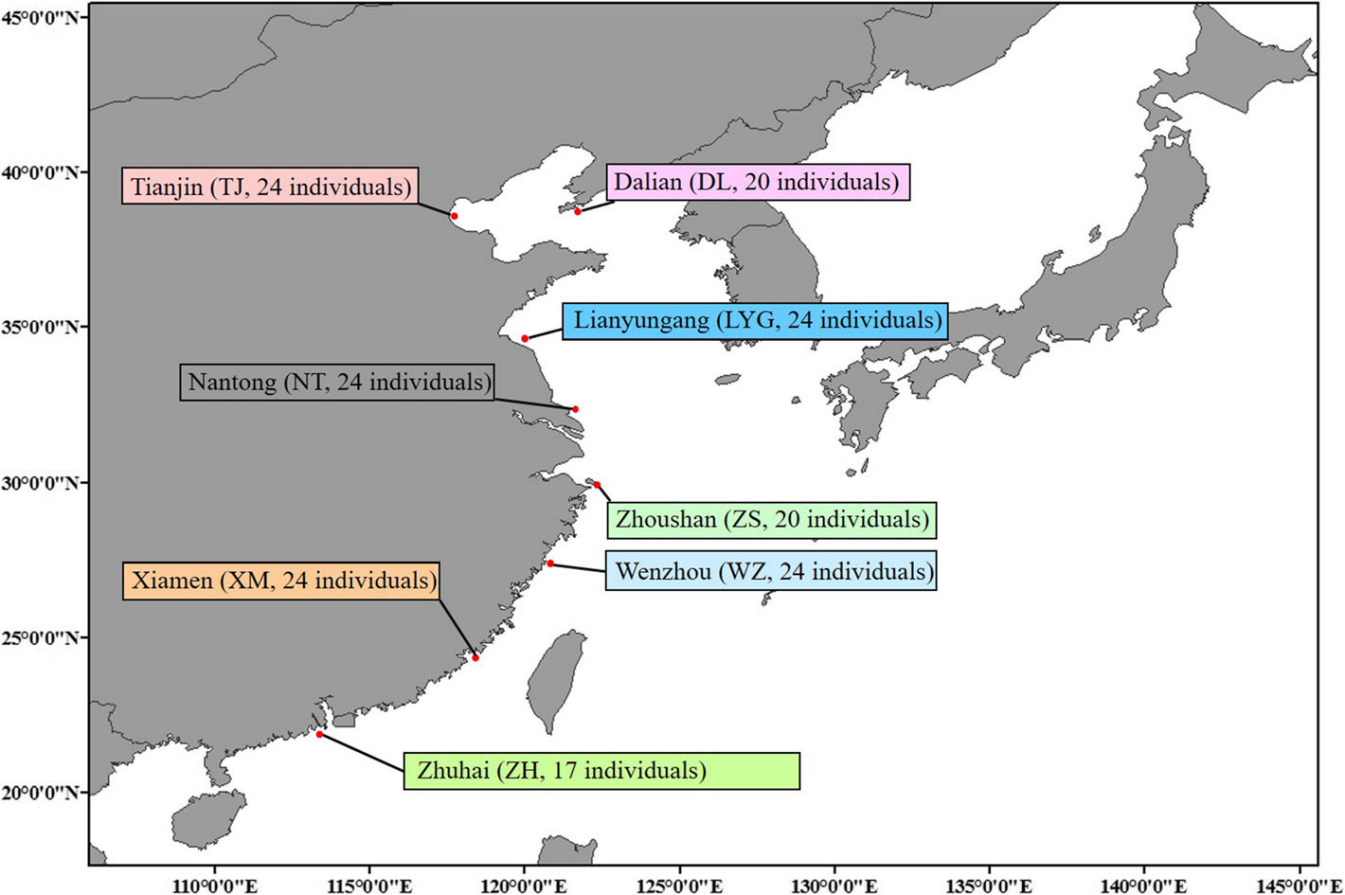
Map of the sampling locations and sample numbers for eight *C. lucidus* populations

In this study, eight *C. lucidus* populations were collected from the coastal waters of China (Fig. 2), and RAD-seq was used to identify genome-wide single nucleotide polymorphisms (SNPs) in the species. Genome-wide SNPs were further used to explore the high-resolution population genetic structure and local adaptation mechanism of *C. lucidus*. This study provides insights into the evolutionary history and genetic diversity of *C. lucidus*. The management units are defined using the temperature-driven SNPs and provide fundamental information for the management of *C. lucidus* resources under fishing pressure.

## Results

### RAD sequencing and genotyping

RAD sequencing of 177 *C. lucidus* individuals resulted in 2,773,840,982 read pairs, 2,707,124,768 of which were retained after quality filtering (Additional file 1). A total of 2,561,795,216 read pairs were properly mapped to the *C. lucidus* genome (GCA_004119915.2; https://www.ncbi.nlm.nih.gov/assembly/GCA_004119915.2) for SNP calling (Additional file 1). After filtering out the low-quality SNPs, 184,708 SNPs were retained for subsequent analyses.

### Population genetic diversity and structure

**Fig. 3:**
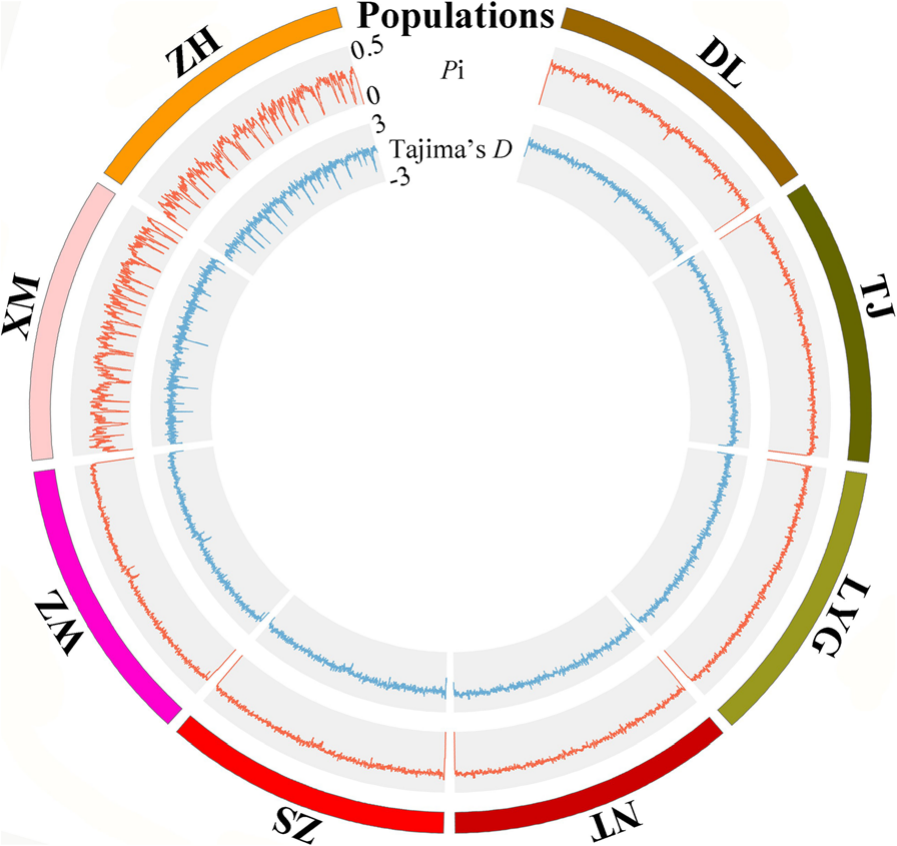
Genome-wide distribution of *P*i and Tajima’s *D* values across eight *C. lucidus* populations based on 184,708 SNPs

These 184,708 SNPs were applied to calculate the genetic diversity of eight *C. lucidus* populations (Table 1). Estimates of observed heterozygosity (*H*_O_), expected heterozygosity (*H*_E_), and polymorphism information (*P*i) averages over the 184,708 SNPs varied across the eight *C. lucidus* populations (*H*_O_ = 0.2353 ~ 0.4147, *H*_E_ = 0.2279 ~ 0.3486, and *P*i = 0.2348 ~ 0.3560). Among the eight *C. lucidus* populations, the TJ and ZH populations showed the highest and lowest *H*_O_, *H*_E_, and *P*i values, indicating the highest and lowest population genetic diversity, respectively. All eight *C. lucidus* populations generally had a high percentage of polymorphic loci (73.4023 ~ 99.4640%). However, the genetic diversity in the XM and ZH populations was relatively low according to all indexes. We also found that the inbreeding coefficient (*F*_IS_) values of the eight *C. lucidus* populations were low (− 0.1477 ~ 0.0209), suggesting that each population contained a large number of individuals. Additionally, most of the pairwise fixation index (*F*_ST_) values between the eight *C. lucidus* populations were significant (Table 2), ranging from 0.00087 to 0.16222. Across all eight *C. lucidus* populations except the XM and ZH populations, the *P*i and Tajima’s *D* values of genome-wide SNPs showed slight fluctuation trends (Fig. 3). The XM and ZH populations showed negative Tajima’s *D* values, while a large proportion of SNPs had positive Tajima’s *D* values in the other populations.

**Table 1:** Genetic diversity statistics of eight *C. lucidus* populations

Populations	Variant Sites	% Polymorphic Loci	Num Indiv	*H*_O_	*H*_E_	*P*i	*F*_IS_
DL	184,708	97.4544	20.0000	0.3460	0.3282	0.3366	−0.0221
TJ	184,708	99.1809	24.0000	0.4147	0.3486	0.3560	−0.1419
LYG	184,708	99.1820	24.0000	0.3353	0.3360	0.3432	0.0209
NT	184,708	99.3254	24.0000	0.3360	0.3358	0.3430	0.0183
ZS	184,708	99.1554	20.0000	0.3440	0.3339	0.3425	−0.0019
WZ	184,708	99.4640	24.0000	0.4118	0.3437	0.3510	−0.1477
XM	184,708	73.4023	24.0000	0.3086	0.2414	0.2465	−0.1442
ZH	184,708	73.6021	17.0000	0.2353	0.2279	0.2348	0.0013

**Table 2:** Pairwise *F*_ST_ values (below diagonal) and *p* values (above diagonal) between eight *C. lucidus* populations

Populations	DL	TJ	LYG	NT	ZS	WZ	XM	ZH
DL	–	**0.00000**	**0.00000**	**0.00000**	**0.00000**	**0.00000**	**0.00000**	**0.00000**
TJ	0.01696	–	**0.00000**	**0.00000**	**0.00000**	**0.00000**	**0.00000**	**0.00000**
LYG	0.01449	0.00268	–	0.12613	**0.00000**	**0.00000**	**0.00000**	**0.00000**
NT	0.01693	0.00262	0.00087	–	**0.00000**	**0.00000**	**0.00000**	**0.00000**
ZS	0.02554	0.00628	0.00598	0.00583	–	**0.00000**	**0.00000**	**0.00000**
WZ	0.02717	0.00601	0.00681	0.00477	0.00434	–	**0.00000**	**0.00000**
XM	0.16222	0.14225	0.14439	0.14089	0.14124	0.13591	–	**0.00000**
ZH	0.16315	0.14313	0.14534	0.14209	0.14195	0.13694	0.00362	–

**Fig. 4:**
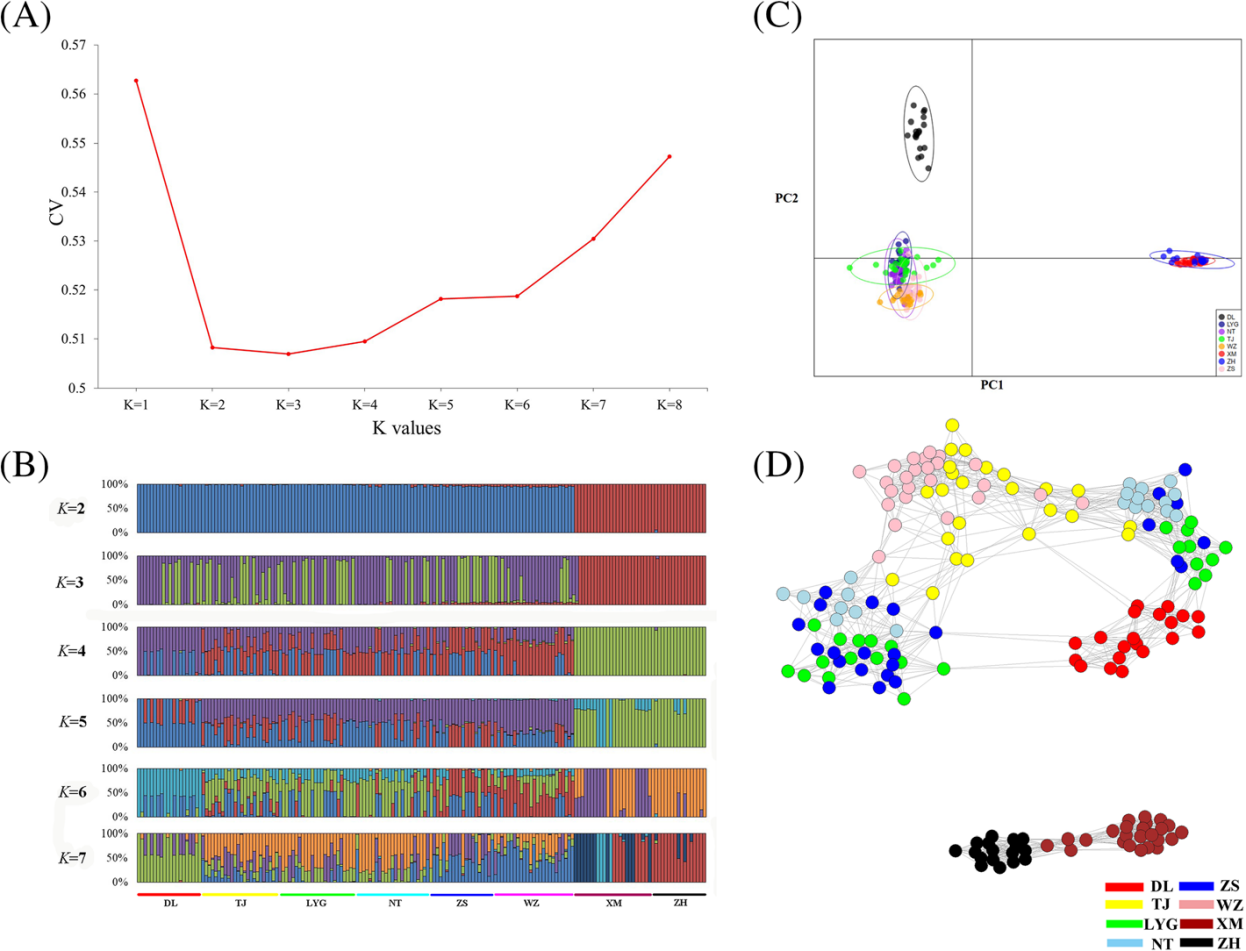
Population structure analysis. (A). CV plot for the Admixture analysis. (B). Plots of *C. lucidus* individual ancestry inference for *K* = 1 to 8 based on 184,708 SNPs. (C). PCA scatter plots with prior population information using first and second components. (D). Individual clustering plot based on NetView P with KNN = 20

ADMIXTURE software was first used for clustering analysis of the eight *C. lucidus* populations. With *K* values of 2 and 3 (Fig. 4A), the DL, TJ, LYG, NT, ZS, and WZ populations formed a cluster, and the XM and ZH populations formed another cluster (Fig. 4B). Meanwhile, the results of the principal component analysis (PCA) were consistent with the ADMIXTURE results (Fig. 4C), which indicated that all populations formed two distinct clusters. NetView P with K-nearest neighbour (kNN) = 20 was applied to reveal the clustering relationships of all *C. lucidus* individuals at a fine scale, and the results further supported the previous ADMIXTURE clustering pattern with *K* = 2 and 3, showing that individuals were grouped into two different clusters, with all individuals from Xiamen and Zhuhai clustered together (Fig. 4D). Additionally, hierarchical AMOVA (Table 3) showed that the *F*_ST_ across the eight populations was 0. 07084, and there was significant genetic differentiation between the two groups (“DL, TJ, LYG, NT, ZS, and WZ” and “XM and ZH”, *F*_CT_ = 0.13, *P* = 0.0004).

**Table 3:** Analysis of molecular variance (AMOVA) performed for two *C. lucidus* groups

Source of Variation	Sum of Squares	Variance Components	Percentage Variation	Fixation Index
Between two groups	590,224.76	4353.42 Va	12.68	*F*_CT_ = 0.13
Among populations within two groups	252,582.39	331.23 Vb	0.96	*F*_SC_ = 0.01
Within eight populations	5,647,712.50	31,907.98 Vc	93.13	*F*_ST_ = 0. 07

### Candidate genomic regions under temperature-driven selection

**Fig. 5:**
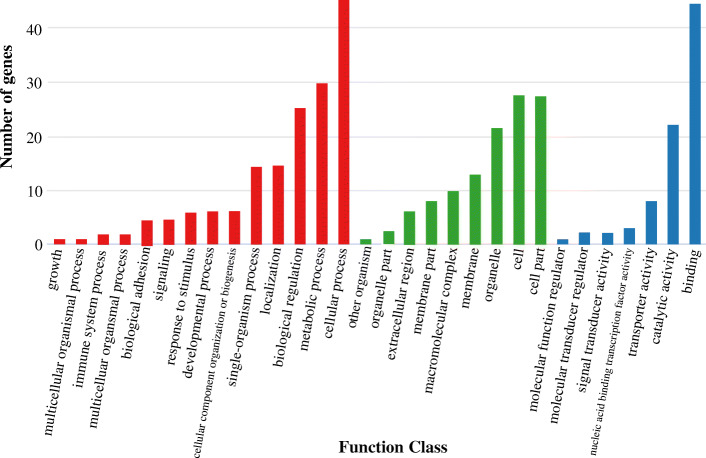
GO annotation information for gene sequences containing temperature-selected SNPs

In the present study, we first calculated the average sea surface temperature (ASST), low sea surface temperature (LSST) and high sea surface temperature (HSST) of the eight sea areas (DL, TJ, LYG, NT, ZS, WZ, XM, and ZH) over 68 years (Additional file 1). Then, Bayenv software revealed a total of 314 SNPs associated with temperature variables. Of these SNPs, 255 were associated with ASST, 56 were associated with LSST, and 4 were associated with HSST. There were 314 union sets among SNPs associated with ASST, LSST and HSST and we used the 314 union sets of ASST-related, LSST-related, and HSST-related SNPs as the candidate temperature-selected SNPs. Of the 314 outlier SNPs, 174 were directly located in intergenic region. The others were aligned to gene region, and only 10 SNPs were assigned to exonic region, which might directly affects protein coding. Gene sequences containing 314 SNPs were then used for further annotations, and the results showed that 105 sequences containing temperature-selected SNPs matched homologous protein sequences in the nonredundant protein sequences (Nr) database (Additional file 1). Next, the enrichment of sequences containing temperature-selected SNPs in Gene Ontology (GO) categories and Kyoto Encyclopedia of Genes and Genomes (KEGG) pathways was tested, and 30 significantly enriched GO terms (Fig. 5) and 16 significantly enriched KEGG pathways (Additional file 1) were identified.

## Discussion

Habitat heterogeneity has profound effects on the population genetic diversity of most marine species. Genome-level analysis not only provides detailed information on the structure, dynamics, and environmental adaptation processes of different populations but also helps us predict how populations will respond to future temperature increases. We first used RAD-seq to obtain genome-wide SNPs in *C. lucidus* and then delineated fine population genetic characteristics and local adaptation characteristics of the eight *C. lucidus* populations at the genomic level. The complete RAD-seq data and hierarchical analysis of variance based on genome-wide SNPs are helpful in providing a reference for population genomics analysis of other marine species and the fine population genetic characteristics based on genome-wide SNPs contributing to the adaptive evolution of *C. lucidus*, especially in the context of ocean temperature rising.

### Genome-wide SNPs delineating the fine population structure of the eight *C. lucidus* populations

Reports of the genetic differentiation of *C. lucidus* are quite limited. The most recent population genetic study was based on the mitochondrial control region [11], which identified *C. lucidus* in offshore China as being divided into a southern group and a northern group, with Zhoushan as the boundary. Song et al. [9] obtained a contradictory conclusion based on a mitochondrial sequence and suggested that the *C. lucidus* in offshore China could be divided into the East China Sea group and the South China Sea group. Herein, we used 184,708 SNPs to provide a higher-resolution analysis of population structure than the abovementioned studies and suggested that *C. lucidus* in offshore China could be divided into northern (DL, TJ, LYG, NT, ZS, and WZ populations) and southern (XM and ZH populations) groups (Fig. 4). This distribution pattern is interesting because there are no obvious barriers between Xiamen and Wenzhou. In fact, the same distribution pattern was also found in *Chelon haematocheilus* [14]. We hypothesize that the reason for the significant genetic differentiation in this distribution pattern as follows: the late Quaternary period (the past one million years) was characterized by a series of large glacial-interglacial changes, and the geographical distributions and abundance of *C. lucidus* may be acutely affected by cycles of sea level rise-and-fall. We have suspected that the decline in sea levels during the glacial period may have led to long-term geographic isolation between the two *C. lucidus* clusters [15] and eventually intensified the development of limited dispersal potential, reproductive isolation and local adaptive heterogeneity between the two clusters [9]. During interglacial periods, although rising sea levels enhanced the dispersal of the two clusters [16, 17], the individuals in the two clusters may not have been able to reproduce, or the diffused individuals may have been unable to adapt to the heterogeneous environment, in turn eventually dying. Over time, significant genetic differentiation between the north and south groups developed.

We also calculated low pairwise *F*_ST_ values within *C. lucidus* clusters, although the *F*_ST_ values between the two *C. lucidus* clusters were high (Table 3). This may prove that the genetic differentiation between the northern and southern groups was more obvious. Meanwhile, the *F*_ST_ values between the two *C. lucidus* clusters probably because that the long-term geographic isolation between the two *C. lucidus* clusters in the glacial period may have contributed to the emergence of endemic adaptive genotypes, and may have limited gene communication between the two groups (Moody et al. 2015). Additionally, we speculate that the pelagic eggs may be a key driver of the low pairwise *F*_ST_ values within *C. lucidus* clusters (Table 3). In fact, ocean current transport can enhance the diffusion ability of the pelagic eggs of *C. lucidus* and therefore significantly increase gene flow within *C. lucidus* clusters, ultimately contributing to the low pairwise *F*_ST_ values within *C. lucidus* clusters [18].

### Genomic regions of temperature selection in eight *C. lucidus* populations

With the advantages of identifying adaptive SNPs, RAD-seq can facilitate insights into the genetics of local adaptation in natural populations [19]. The *C. lucidus* samples in this study were collected from eight different geographical locations and temperature may be one of the important selective forces affecting the genotypic and phenotypic compositions of local populations. In the present study, ASST, LSST and HSST at eight sampling sites were used to identify temperature-selected SNPS and different numbers of candidate SNPs were obtained using different datasets (ASST, LSST, and HSST). We have suspected that those important candidate SNPs could be affected by temperature heterogeneity.

The GO annotation results showed that the sequences containing the temperature-selected SNPs were mainly involved in metabolic and cellular processes, and their functions were mainly in binding and catalytic activity (Fig. 5). This suggests that temperature differences may drive adaptive differentiation in parts of the genome of *C. lucidus* populations, ultimately leading to differences in physiological regulation. The KEGG annotation results showed that the sequences containing temperature-selected SNPs were mainly associated with material (protein, lipid, and carbohydrate) metabolism and immune responses (Additional file 1). A previous study revealed that the metabolic capacity of organisms was more susceptible to selection under selective force [20]. Moreover, proteins, lipids, and carbohydrates are critical energy materials [21]. Additionally, Lou et al. [22] hypothesized that temperature can affect the priority of metabolic patterns, such as anaerobic carbohydrate metabolism, which is preferred by organisms at low temperatures. Therefore, we speculate that mutations in material metabolism-related genes may cause different *C. lucidus* populations to change their metabolic patterns in order to maintain a maximum metabolic capacity at different temperatures. Additionally, environmental heterogeneity will inevitably cause variation in environmental pressure among *C. lucidus* populations. Mutations in genes associated with the immune response may provide evidence of resistance specificity to temperature stress in different geographic populations (Additional file 1). For example, the immune response was shown to be related to local adaptation to different water temperatures in *Tylosurus crocodilus crocodilus* [23], *Trachidermus fasciatus* [13], and *Larimichthys polyactis* [24].

In our outlier study, only *RAR-α* (Retinoic acid receptor alpha) gene was found to be associated with HSST (Additional file 1). Previous studies have considered that *RAR-α* gene is involved in the regulation of cell differentiation, proliferation, apoptosis, embryonic development, visual formation, bone formation, metabolism, haematopoiesis and many other pivotal biological processes [25]. Compared with HSST, more SNPs were associated with LSST, which suggests that low temperature pressure may have more serious effects on the spatial distribution of *C. lucidus*. Poikilotherms have been shown to alter the substance metabolism to maximize ATP production efficiency at ambient temperatures [26]. In our LSST-related outlier study, several genes associated with material metabolic functions were annotated (Additional file 1). For instance, a gene (glucagon-1-like, *GLR-1*) appears to stimulate insulin production and suppress glucagon production [27]. Therefore, we hypothesize that genes associated with glycometabolism provide energy for cell survival and ultimately increase the ability of different *C. lucidus* populations to survive in low temperatures. Lower seawater temperatures may enhance oxidative stress of marine organisms. In the present study, we found that some genes were associated with oxidative stress (Additional file 1), such as *TLR9* (toll-like receptor 9). *TLR9* gene is one of pattern recognition receptors (PRRs). As the sensory receptors of organisms to environmental stress, PRRs has been proved to play an important role in innate immune response [28]. Previous studies have showed that Na^+^ and Cl^−^ account for a relatively major proportion of the composition of haemolymph in most marine organisms [29]. Our results showed that several LSST-related SNPs are located in ion transport-related genes (*SLC6A5*; sodium- and chloride-dependent glycine transporter 2), and these genes may influence the sensitivity of different *C. lucidus* populations to Na^+^ and Cl^−^ [30]. In fact, the salinity of the low-temperature areas offshore China is higher than that of the high-temperature areas, therefore, we suspected that different *C. lucidus* populations have evolved different osmotic pressure regulation abilities. Interestingly, one gene (*F9*; coagulation factor IX) containing LSST-related SNP has been annotated to be involved in the coagulation process. Moderate hypothermia has been used as one of the international anti-coagulant methods [31], the mutations in the *F9* gene may has reduces the coagulation rates of *C. lucidus* populations in the low temperatures.

In summary, the temperature-selected SNPs analysis revealed a number of potential genes that help different *C. lucidus* populations adapt to local temperatures. However, it is undeniable that some temperature-selected SNPs could be false positives. Meanwhile, future studies will also need to conduct experiments (i.e. RNA-seq, gene knockdown or genome editing) to investigate the exact function and expression levels of these candidate genes.

## Conclusion

In the present study, RAD-seq was applied to develop the genome-wide SNPs of *C. lucidus*. All SNPs were further used to reveal the population genetic structure and genomic regions under temperature-driven selection in eight *C. lucidus* populations. Genetic structure analysis showed that there was significant genetic differentiation between the northerm (DL, TJ, LYG, NT, ZS, and WZ populations) and southern (XM and ZH populations) groups. Combined with the temperature data of eight sea areas (DL, TJ, LYG, NT, ZS, WZ, XM, and ZH) over 68 years, 314 SNPs associated with temperature variables were identified and genes that contained temperature-selected SNPs were involved in material (protein, lipid, and carbohydrate) metabolism and immune responses. The present research could help us determine how different *C. lucidus* populations respond to local temperatures.

## Methods

### Specimen collection and RAD sequencing

All *C. lucidus* samples were collected from eight locations in China, namely, Dalian (DL), Tianjin (TJ), Lianyungang (LYG), Nantong (NT), Wenzhou (WZ), Zhoushan (ZS), Xiamen (XM), and Zhuhai (ZH) (Fig. 2 and Additional file 1). Muscles were extracted from each individual using sterilized scissors and forceps. All muscle tissues were separately preserved in 95% ethanol and stored at − 80 °C prior to the subsequent experiments. Genomic DNA was extracted following the standard phenol-chloroform extraction method. After assessing the quality of the genomic DNA, we constructed a paired-end library with high-quality genomic DNA following the protocol described by Etter et al. [32] and then sequenced the library on the Illumina HiSeq 2500 sequencing platform.

### RAD data processing and SNP filtering

All raw reads in FASTQ format were filtered using Trimmomatic software (version 0.36 [33];) based on the following criteria: (I) raw reads with sequencing adaptors; (II) a ratio of unidentified nucleotides in the raw reads ≥8%; and (III) raw reads that had more than 50% of base calls with a low quality score (Q < 30). After filtering, we downloaded the whole-genome sequence of *C. lucidus* [34] and used it as a reference sequence for subsequent SNP filtering. The whole-genome sequence was first constructed into an index file using BWA software (version 0.7.12 [35];). The clean reads of each sample were then aligned to the whole-genome sequence using the “bwa-mem” algorithm in BWA software (version 0.7.12 [35];) with default parameters. SNP calling was subsequently performed using SAMtools software (version 1.3.1 [36];) with the following parameters: -q 1 -C 50 -t AD, ADF, ADR, DP, SP -m 2 -F 0.002. The generated SNPs were sorted in a variant call format (VCF) file. Furthermore, we removed the low-quality SNPs using SAMtools software (version 1.3.1 [36];) with the following parameters: --maf 0.01 --max-missing 0.1 --min-meanDP 150 --min-alleles 2 --max-alleles 2 --minGQ 98 --minQ 30 --remove-indels --hwe 0.05.

### Population genetic diversity and differentiation

The genome-wide patterns of genetic variation, including nucleotide diversity (*P*i) and Tajima’s *D* at each SNP, were estimated using TASSEL software (version 5.2.31 [37];). The results were visualized using Circos software [38]. To calculate the genetic diversity within populations, nucleotide diversity (*P*i), observed heterozygosity (*H*_O_), expected heterozygosity (*H*_E_), and the inbreeding coefficient (*F*_IS_) were calculated using the “populations” module in Stacks software (version 1.34 [39];. Pairwise genetic differentiation (*F*_ST_) values between populations and their significance were calculated using Arlequin software (version 3.5 [40]; with 10,000 permutations.

Population genetic structure based on all SNPs was analysed using five methods. (I) The Bayesian model-based clustering program ADMIXTURE (version 1.3.0 [41];) was used to investigate individual ancestries, with five replicates of coancestry cluster (*K*) values ranging from 1 to 8. The optimal *K* value corresponded to the lowest cross-validation error. (II) We conducted a principal component analysis (PCA) using the “*adegenet*” package [42] implemented in R software to infer population structure. (III) We calculated the allele-sharing distance using PLINK software [43]. The NetView pipeline (version 0.7.1 [44];) with a KNN step ranging from 1 to 45 was then used to construct the fine-scale relationships between all individuals, and the networks were visualized using Cytoscape software [45]. (IV) On the basis of the optimal value of *K*, we divided all the groups into two groups (group 1: DL, TJ, LYG, NT, ZS, and WZ; group 2: XM and ZH) and then performed an analysis of molecular variance (AMOVA) in Arlequin software (version 3.5 [40];) to estimate the differentiation among groups (*F*_CT_) and the differentiation among populations within groups (*F*_SC_).

### Outlier SNP detection and annotation

The genotype-environment association method implemented in Bayenv (version 2.0 [46];) was applied to detect putative SNPs correlated with temperature variations. First, we obtained the high-resolution mean LSST, mean ASST and mean HSST data of eight sea areas (DL, TJ, LYG, NT, ZS, WZ, XM, and ZH) over 68 years (from 1950 to 2017) by combining data from the Japan Meteorological Agency (JMA; http://www.jma.go.jp/jma/index.html), Advanced Very High Resolution Radiometer (AVHRR; http://oceanwatch.pifsc.noaa.gov/thredds/catalog.html) and Geostationary Operational Environmental Satellites (GOES; http://oceanwatch.pifsc.noaa.gov/thredds/catalog.html). We first converted SNPs files and environmental data files into matrix files needed for software operation and the matrix was evaluated according to the software specification, the parameters were set as follows: -k 100,000 -r 63,479. Then, Bayenv tests were applied to identify putative SNPs correlated with temperature variations using “calc_bfs.sh” script in the Bayenv2 software, and a Bayes factor (BF) value higher than 10 was set as the filtering condition for putative SNPs. We repeated the Bayenv analysis four times to avoid false positives, and only the SNPs that were continuously screened were used for subsequent analysis. Thereafter, we used the union set of ASST-related, LSST-related, and HSST-related SNPs as the candidate temperature-selected SNPs. To determine the genetic mechanisms underlying temperature-related adaptive differentiation between *C. lucidus* populations, gene sequences containing these SNPs were then annotated using Blast2GO software along with KEGG database [47, 48]. Meanwhile, for identified the outlier SNPs in genome, we used ANNOVAR software [49] to annotate them based on genome annotation information of *C. lucidus* (GCA_004119915.2).

## Supplementary Information

**Additional file 1: Supplementary tables**

## Data Availability

Sequences are available from GenBank with the Bioproject accession number PRJNA679902 (https://www.ncbi.nlm.nih.gov/bioproject/PRJNA679902).

## Abbreviations

*H*_O_: Observed heterozygosity
*H*_E_: Expected heterozygosity
*P*i: Polymorphism information;
*F*_IS_: The inbreeding coefficient
*F*_ST_: Fixation index
PCA: Principle component analysis
kNN: K-nearest neighbor
ASST: Average sea surface temperature
LSST: Low sea surface temperature
HSST: High sea surface temperature
