# Supplementary material for: Population genomics provides insights into the population structure and temperature-driven adaptation of Collichthys lucidus
Source: BMC Genomics. 2021 Oct 8;22:729. doi: 10.1186/s12864-021-08045-8 (PMC8501621; doi:10.1186/s12864-021-08045-8)
Supplement: Supplementary file 1 — Additional file 1: Supplementary tables [file 12864_2021_8045_MOESM1_ESM.pdf]

Population genomics provides insights into the population structure and temperature-driven adaptation of *Collichthys lucidus*

Linlin Zhao[1], Fangyuan Qu[1], Na Song[2], Zhiqiang Han[3], Tianxiang Gao[3], Zhaohui Zhang[1*]

[1] First Institute of Oceanography, Ministry of Natural Resources, Qingdao, Shandong, 266100, China.

[2] Fishery College, Ocean University of China, Qingdao, Shandong, 266061, China.

[3] Fishery College, Zhejiang Ocean University, Zhoushan, Zhejiang, 316022, China.

*Corresponding author: zhang@fio.org.cn

Supplementary tables

Table S1. Sequence information for all individuals.

Population code	Individual ID	Total reads	Filtered reads	Mapped paired reads
DL	DL1	12,144,705	12,036,546	11,394,594
DL	DL2	42,739,188	42,375,199	39,985,776
DL	DL3	17,363,571	17,219,734	16,361,906
DL	DL6	7,858,564	7,796,652	7,394,432
DL	DL7	26,409,626	26,197,108	24,792,344
DL	DL8	56,987,595	56,574,671	53,529,488
DL	DL9	17,604,000	17,431,088	16,528,280
DL	DL10	19971341	19806084	18544348
DL	DL11	16072879	15942616	15078908
DL	DL12	11132757	11029476	10441068
DL	DL13	7085705	7020131	6637064
DL	DL15	14564901	14462622	13692528
DL	DL16	9803928	9734219	9158470
DL	DL17	7250697	7195839	6847992
DL	DL18	12652424	12541618	11920028
DL	DL19	16662581	16506847	15683814
DL	DL20	12114619	12017397	11398322
DL	DL22	9035525	8956415	8512848
DL	DL23	15478690	15326882	14447016
DL	DL24	7818329	7765875	7368030

Population code	Individual ID	Total reads	Filtered reads	Mapped paired reads
TJ	TJ10	10398295	10026387	9372868
TJ	TJ11	16244585	15368691	14377658
TJ	TJ12	16154170	15668974	14702228
TJ	TJ13	12224786	11870828	11072596
TJ	TJ14	11345864	10128549	9465648
TJ	TJ15	15496082	12094604	11349726
TJ	TJ16	15400721	14989040	13947006
TJ	TJ17	17361594	16519677	15513078
TJ	TJ18	22330790	20755565	19514408
TJ	TJ19	50670544	49714315	46847062
TJ	TJ1	19027861	18078921	16979746
TJ	TJ20	14847069	14245590	13378718
TJ	TJ21	11123168	3901538	3675414
TJ	TJ22	15234256	14337597	13426368
TJ	TJ23	12186054	11148600	10474322
TJ	TJ24	14490523	13258247	12454016
TJ	TJ2	13690324	12513129	11735784
TJ	TJ3	22454634	21626796	20291392
TJ	TJ4	13123169	12107625	11319140
TJ	TJ5	11054419	10639828	9968906
TJ	TJ6	17539598	17107366	16061196
TJ	TJ7	11738557	11233328	10556396
TJ	TJ8	14099113	13639635	12760042
TJ	TJ9	20270494	19415663	18158260
LYG	LYG1	15138384	14902953	14077386

Population code	Individual ID	Total reads	Filtered reads	Mapped paired reads
LYG	LYG2	11904889	11747570	11053506
LYG	LYG3	10453910	10338559	9760254
LYG	LYG4	16990856	16803321	15942530
LYG	LYG5	16558166	16399444	15541050
LYG	LYG6	16321753	16137519	15308460
LYG	LYG7	12097145	11981825	11355178
LYG	LYG8	18596419	18251946	17195728
LYG	LYG9	11816634	11221658	10644968
LYG	LYG10	14400949	13990429	13245096
LYG	LYG11	13082316	12721972	12013576
LYG	LYG12	13870100	13655298	12878432
LYG	LYG13	10941291	10672689	10126132
LYG	LYG14	12504181	12383053	11782284
LYG	LYG15	16210450	15984076	15099760
LYG	LYG16	12711351	12548287	12047930
LYG	LYG17	13900422	13769403	13134056
LYG	LYG18	13338339	13189501	12491260
LYG	LYG19	11234559	10993384	10344642
LYG	LYG20	18981369	18820200	17775550
LYG	LYG21	11507451	11313115	10837890
LYG	LYG22	12492275	12321395	11622200
LYG	LYG23	11150397	11008608	10399722
LYG	LYG24	16894364	16658442	15747810
NT	NT1	13390754	13192269	12420662
NT	NT2	14553769	14351203	13580476

Population code	Individual ID	Total reads	Filtered reads	Mapped paired reads
NT	NT3	19478188	19230067	18192604
NT	NT4	13889686	13709091	12949686
NT	NT5	16649337	16464382	15508070
NT	NT6	29838951	27850782	26478400
NT	NT7	14706170	11935239	11274174
NT	NT8	13567167	13428082	12546324
NT	NT9	19215810	19026972	17990278
NT	NT10	15824139	15677585	14682236
NT	NT11	10006371	9806436	9167678
NT	NT12	19927083	18939932	17860818
NT	NT13	12135314	11955948	11276800
NT	NT14	16660694	16470447	15557674
NT	NT15	15313061	15122206	14298762
NT	NT16	12254083	12120380	11429920
NT	NT17	17391336	16875579	15958516
NT	NT18	10523382	10363662	9774920
NT	NT19	17518499	17084401	16022278
NT	NT20	19202769	19000001	18041854
NT	NT21	15395410	15160671	14337504
NT	NT22	12102944	11961114	11249662
NT	NT23	16747886	16232925	15378616
NT	NT24	14930008	14734856	13901338
ZS	ZS10	8136445	8088546	7683998
ZS	ZS11	8435796	8383411	7953668
ZS	ZS13	8139324	8091535	7669156

Population code	Individual ID	Total reads	Filtered reads	Mapped paired reads
ZS	ZS14	7689142	7632722	7230968
ZS	ZS16	8051306	8003476	7584566
ZS	ZS17	6399906	6359991	6029758
ZS	ZS18	8111785	7934409	7460226
ZS	ZS19	15542467	15341866	14530744
ZS	ZS1	18008972	17897061	16970642
ZS	ZS21	12060871	11945250	11298102
ZS	ZS23	23234006	22985656	21766832
ZS	ZS24	24311016	24120599	22867052
ZS	ZS2	22724167	22394105	21270708
ZS	ZS3	20626330	20493549	19411754
ZS	ZS4	8754044	8703418	8275356
ZS	ZS5	27198960	27028827	25676170
ZS	ZS6	12107490	12037267	11443500
ZS	ZS7	24111084	23950158	22714008
ZS	ZS8	25332473	25172199	23926578
ZS	ZS9	15208352	15098762	14297492
WZ	WZ10	13964421	13721652	12806042
WZ	WZ11	20871452	20533812	19314750
WZ	WZ12	19703791	19339508	18133168
WZ	WZ13	12755768	12459980	11655118
WZ	WZ14	11524644	11362590	10595538
WZ	WZ15	16140413	15893294	14932692
WZ	WZ16	10489762	10366116	9643188
WZ	WZ17	10944033	10798696	10080006

Population code	Individual ID	Total reads	Filtered reads	Mapped paired reads
WZ	WZ18	18338365	17888747	16825588
WZ	WZ19	22175454	21833325	20560108
WZ	WZ1	15628817	15358515	14358630
WZ	WZ20	14810439	14599069	13702940
WZ	WZ21	13081577	12824356	12086678
WZ	WZ22	20078470	19706449	18578370
WZ	WZ23	25665000	25046488	23688292
WZ	WZ24	22834670	22336498	21096420
WZ	WZ2	16479421	16220689	15198438
WZ	WZ3	26439961	25905668	24222406
WZ	WZ4	16715575	16435394	15438836
WZ	WZ5	17779914	17409623	16378372
WZ	WZ6	13818891	13360351	12564468
WZ	WZ7	35608172	34895998	32972214
WZ	WZ8	16149453	15821330	14868238
WZ	WZ9	18771330	18369217	17255240
XM	XM10	12973475	12823631	12229800
XM	XM11	15106709	14909071	14265418
XM	XM12	11934351	11745614	11268242
XM	XM13	16893732	15837479	14959750
XM	XM14	16262177	15985870	15105656
XM	XM15	22461906	22003279	20906332
XM	XM16	14279996	14043348	13288640
XM	XM17	17473136	17268662	16509776
XM	XM18	12927399	12670317	12144350

Population code	Individual ID	Total reads	Filtered reads	Mapped paired reads
XM	XM19	23278750	22909905	21990592
XM	XM1	12941196	12731981	12142306
XM	XM20	13533646	13317807	12770652
XM	XM21	11545886	11360503	10887840
XM	XM22	17345958	17112374	16382376
XM	XM23	18386687	17903054	17218864
XM	XM24	10514315	10228692	9844224
XM	XM2	15457426	15235662	14514082
XM	XM3	11900167	11660112	11142802
XM	XM4	13114867	12941775	12296100
XM	XM5	11074558	10921451	10428202
XM	XM6	13125160	12932532	12357402
XM	XM7	12697880	12506877	12002418
XM	XM8	10458932	10326942	9893016
XM	XM9	12177059	11966335	11478220
ZH	ZH10	15783416	15432901	14818788
ZH	ZH11	13559768	13359052	12809814
ZH	ZH13	15387031	15260876	14669920
ZH	ZH14	11013328	10920381	10474526
ZH	ZH17	14480910	14203389	13618978
ZH	ZH18	14422667	14308936	13768698
ZH	ZH20	9273561	9189701	8823190
ZH	ZH21	16604343	16459388	15765930
ZH	ZH22	9510104	9440188	9058336
ZH	ZH23	23003085	22706847	21837626

Population code	Individual ID	Total reads	Filtered reads	Mapped paired reads
ZH	ZH24	9994312	9910054	9515056
ZH	ZH2	17070343	16966074	16290954
ZH	ZH3	15503501	15383885	14754120
ZH	ZH4	8476509	8266170	7928322
ZH	ZH6	19096830	18886922	18094456
ZH	ZH7	13197606	13113338	12575280
ZH	ZH9	11065495	10986832	10523260
Total		2773840982	2707124768	2561795216

Table S2. The average ASST, LSST and HSST of eight sea areas.

	ASST (℃)	LSST (℃)	HSST (℃)
DL	12.81	3.18	23.62
TJ	13.12	2.25	24.60
LYG	16.58	8.85	24.51
NT	17.25	8.82	25.07
ZS	19.39	11.19	27.26
WZ	21.00	14.34	27.48
XM	23.02	17.70	27.60
ZH	25.02	20.27	28.91

Table S3. Nr annotation information for the whole genome containing temperature-selective SNPs.

SNPs	Nr_description	#Hits	e-Value	sim mean
SNP_12343	RNA-directed DNA polymerase from mobile element jockey	20	3.71181E-174	74.7
SNP_16585	SCAN domain-containing protein 3-like	16	4.81E-07	79.65
SNP_17991	voltage-dependent calcium channel gamma-1 subunit	20	4.78E-07	94.54
SNP_18949	tether containing UBX domain for GLUT4	20	2.99E-12	81.05
SNP_19487	ral guanine nucleotide dissociation stimulator-like 1 isoform X2	20	5.24E-14	93.02
SNP_22368	deoxyuridine 5'-triphosphate nucleotidohydrolase, mitochondrial isoform X2	20	4.03E-26	80.02
SNP_26947	transcription factor Maf	20	3.14E-49	99.66
SNP_27784	zinc finger homeobox protein 3 isoform X1	20	3.27E-75	96.85
SNP_29094	polycystic kidney disease protein 1-like 2	20	2.44E-31	91.46
SNP_29163	ankyrin repeat domain-containing protein 11 isoform X1	20	1.71E-15	97.51
SNP_30113	UPF0258 protein KIAA1024	5	1.36E-19	88.7
SNP_32180	V-type proton ATPase subunit S1-like	20	1.55E-22	93.72
SNP_36478	RNA-directed DNA polymerase from mobile element jockey	20	9.35E-85	70.64
SNP_36534	calcium-dependent secretion activator 1-like	20	1.48E-16	98.93
SNP_37414	chromodomain-helicase-DNA-binding protein 6	20	7.04E-22	56.58
SNP_37934	forkhead box protein P4 isoform X3	20	4.04E-09	100
SNP_38644	zinc finger protein 385A-like isoform X1	20	6.26E-27	91.88
SNP_39567	uncharacterized protein LOC104921080	20	7.14E-103	94.95
SNP_39969	Transposable element	20	1.06E-61	68.86
SNP_42261	rho guanine nucleotide exchange factor 6 isoform X1	20	7.21E-08	63.48
SNP_43929	hypothetical protein FQN60_010327	1	3.33E-17	58.65
SNP_44195	ras-GEF domain-containing family member 1C isoform X1	20	9.52E-15	95.13
SNP_44893	Protein FAM193B	20	3.00E-81	95.44

SNP_47723	U4/U6 small nuclear ribonucleoprotein	5	4.11E-10	73.76
SNP_48351	zinc finger BED domain-containing protein 1-like	20	4.31E-26	57.23
SNP_50620	protein tyrosine phosphatase domain-containing protein 1-like	20	6.84E-41	98.56
SNP_50940	semaphorin-3D	20	1.12E-14	96.08
SNP_51641	hypothetical protein F2P81_007000	1	2.89E-05	83.33
SNP_55016	U4/U6 small nuclear ribonucleoprotein	2	3.81E-09	79.73
SNP_55301	ataxin-2 homologue	20	4.52E-17	69.93
SNP_56905	tetratricopeptide repeat protein 14 isoform X2	20	7.21E-37	89.31
SNP_58184	cAMP-specific 3',5'-cyclic phosphodiesterase 4C isoform X5	20	1.61E-30	97.19
SNP_58433	peptidyl-tRNA hydrolase ICT1, mitochondrial isoform X1	1	8.47E-04	62.3
SNP_59921	single-stranded DNA-binding protein 3 isoform X2	20	4.14E-11	91.26
SNP_61280	transmembrane protein 44 isoform X1	20	8.28E-37	71.26
SNP_62167	unconventional myosin-VIIa-like	20	3.18E-77	78.13
SNP_65762	kinesin-like protein KIF26B isoform X2	20	0	72.12
SNP_66362	centrosomal protein of 128 kDa isoform X1	20	3.56E-15	91.62
SNP_67013	mitogen-activated protein kinase-binding protein 1-like	3	5.53E-05	88.89
SNP_67838	tyrosine-protein kinase	4	1.56E-22	95.69
SNP_68563	inactive hydroxysteroid dehydrogenase-like protein 1	20	7.97E-28	91.52
SNP_70041	E3 ubiquitin-protein ligase znrf2	2	6.51E-08	74.19
SNP_71633	tumour necrosis factor receptor superfamily member 5-like isoform X1	20	1.06E-50	74.46
SNP_74870	protein bassoon-like	20	6.15E-14	97.51
SNP_74960	monoglyceride lipase isoform X2	20	4.50E-33	96.69
SNP_74961	monoglyceride lipase isoform X2	20	4.50E-33	96.69
SNP_75583	tumour necrosis factor receptor superfamily member 1A-like	20	7.07E-74	89.31
SNP_75600	thymocyte selection-associated high mobility group box protein TOX-like isoform X4	20	3.71E-24	93.46
SNP_76208	structural maintenance of chromosomes protein 5	20	4.89E-43	80.09

SNP_81377	collagen alpha-1(XXVII) chain B	1	5.41E-10	77.78
SNP_81675	nitric oxide synthase, brain	20	1.14E-21	87.82
SNP_83405	hypothetical protein D9C73_023402	1	4.22E-04	88.89
SNP_87055	peroxisomal biogenesis factor 19	20	8.04E-82	66.38
SNP_87913	receptor-type tyrosine-protein phosphatase mu isoform X12	20	1.78E-18	97.96
SNP_90686	histone deacetylase 5	1	4.23E-09	92.11
SNP_92062	F-box-like/WD repeat-containing protein TBL1X	20	2.61E-35	92.98
SNP_93635	catenin delta-2-like	20	4.24E-16	93.97
SNP_93815	acyl-coenzyme A thioesterase 11-like	20	1.39E-86	88.68
SNP_93865	piggyBac transposable element-derived protein 3-like	20	2.02E-31	77.23
SNP_95187	C2 domain-containing protein 3 isoform X1	20	2.82E-69	60.49
SNP_96553	protein Smaug homologue 2	20	1.01E-30	96
SNP_98335	LIM domain kinase 1-like isoform X3	20	1.67E-27	98.41
SNP_108133	DNA-binding death effector domain-containing protein 2	20	1.47149E-111	72.16
SNP_109885	C-type lectin domain family 4 member A-like isoform X1	20	9.10E-44	83.55
SNP_111031	methyltransferase-like protein 6	20	2.12E-49	97.02
SNP_111569	hypothetical protein D9C73_016790	2	1.19E-12	100
SNP_113911	E3 ubiquitin-protein ligase Jade-2	20	8.14E-08	84.55
SNP_114544	cytosolic arginine sensor for mTORC1 subunit 1 isoform X3	20	1.63E-06	72.13
SNP_118917	hypothetical protein D9C73_017765	3	6.87E-24	63.93
SNP_121133	AT-hook-containing transcription factor isoform X1	20	1.42E-19	77.94
SNP_124816	synaptic vesicle 2-related protein	1	4.39E-27	100
SNP_126150	collagen alpha-1(V) chain Precursor	5	3.46E-16	94.59
SNP_126952	ribosomal protein L7a	20	4.10E-33	87.64
SNP_127271	secretory carrier-associated membrane protein 1	20	2.91E-09	93.35
SNP_127448	tyrosine-protein phosphatase non-receptor type 11 isoform X1	20	6.64E-46	59.93

SNP_130027	interferon-induced, double-stranded RNA-activated protein kinase	20	2.25E-59	71.99
SNP_130411	transforming acidic coiled-coil-containing protein 2	2	1.22E-08	73.81
SNP_130412	transforming acidic coiled-coil-containing protein 2	2	1.22E-08	73.81
SNP_130530	cAMP and cAMP-inhibited cGMP 3',5'-cyclic phosphodiesterase 10A	20	1.54E-49	61.57
SNP_130956	dysbindin-A biogenesis of lysosome-related organelles complex 1 subunit 8-A	1	6.31E-05	80
SNP_134330	serine/threonine-protein phosphatase 4 regulatory subunit 3B	20	1.53E-99	100
SNP_138709	collagen alpha-1(XII) chain isoform X1	20	6.66E-51	69.2
SNP_139122	kinesin-like protein KIF26A	1	6.99E-08	100
SNP_139181	transmembrane protein 260 isoform X2	20	5.25E-30	92.95
SNP_139502	leucine-rich repeat-containing protein 74A	20	1.20E-75	60.27
SNP_141194	mitochondrial peptide methionine sulfoxide reductase	20	2.92E-17	96.09
SNP_144618	R2DM retrovirus-related Pol polyprotein from type II retrotransposable element	20	1.15E-123	92.9
SNP_145702	hypothetical protein D4764_12G0007310	11	3.79E-13	58.69
SNP_146433	cytoplasmic protein NCK1 isoform X2	20	8.31E-136	97.28
SNP_146530	solute carrier family 22 member 13-like	20	2.62E-23	84.54
SNP_146620	villin-like protein isoform X2	20	1.18E-52	86.72
SNP_151525	DENN domain-containing protein 3-like	20	2.89E-61	60.88
SNP_151970	probable 2-ketogluconate reductase isoform X1	20	3.94E-71	69.3
SNP_153497	hypothetical protein E3U43_010683, partial	1	3.74E-15	87.23
SNP_155153	protein FAM135B	20	6.12E-26	91.61
SNP_156422	semaphorin-4A-like isoform X1	20	4.64E-67	94.48
SNP_157648	serine protease HTRA1B-like	20	9.95E-22	96.47
SNP_160669	zinc finger MIZ domain-containing protein 1 isoform X1	20	5.36E-25	86.96
SNP_161867	5-hydroxytryptamine receptor 3A-like	20	2.72E-53	62.6
SNP_164454	zinc transporter ZIP11 isoform X2	20	1.02E-11	89.49
SNP_170110	mitochondrial-processing peptidase subunit beta	20	1.51E-10	95.69

SNP_171054	integrin alpha-3-like	20	4.42E-21	89.24
SNP_172213	E3 ubiquitin-protein ligase znrf2	2	1.79E-08	84.75
SNP_178256	T-cell receptor beta-1 chain C region	5	1.94E-27	72.54
SNP_178968	E3 ubiquitin-protein ligase znrf2	2	8.12E-06	80.51

Table S4. KEGG annotation information for the whole genome containing temperature-selective SNPs.

Pathway	Pathway ID	Enzyme
Nicotinate and nicotinamide metabolism	ko00760	ec:3.6.1.9 - diphosphatase
Glyoxylate and dicarboxylate metabolism	ko00630	ec:1.11.1.6 – equilase
Purine metabolism	ko00230	ec:3.6.1.9 - diphosphatase, ec:3.1.4.17 - phosphodiesterase
Phenylpropanoid biosynthesis	ko00940	ec:1.11.1.7 - lactoperoxidase
PD-L1 expression and PD-1 checkpoint pathway in cancer	ko05235	ec:3.1.3.16 - phosphatase
Tryptophan metabolism	ko00380	ec:1.11.1.6 - equilase
Starch and sucrose metabolism	ko00500	ec:3.6.1.9 - diphosphatase
Drug metabolism - other enzymes	ko00983	ec:3.6.1.23 - diphosphatase
Arginine biosynthesis	ko00220	ec:1.14.13.39 - synthase (NADPH)
Th1 and Th2 cell differentiation	ko04658	ec:3.1.3.16 - phosphatase
T cell receptor signalling pathway	ko04660	ec:2.7.10.2 - protein-tyrosine kinase, ec:3.1.3.16 - phosphatase
Riboflavin metabolism	ko00740	ec:3.6.1.9 - diphosphatase
Pyrimidine metabolism	ko00240	ec:3.6.1.9 - diphosphatase, ec:3.6.1.23 - diphosphatase
Pantothenate and CoA biosynthesis	ko00770	ec:3.6.1.9 - diphosphatase
Arginine and proline metabolism	ko00330	ec:1.14.13.39 - synthase (NADPH)
Glycerophospholipid metabolism	ko00564	ec:2.7.8.5 - 1-phosphatidyltransferase

Table S5. Sampling information for eight sites of *C. lucidus*.

Site	Code	Samples	Latitude	Longitude	Year
Dalian	DL	20	38.88	121.82	2017
Tianjin	TJ	24	38.82	117.74	2018
Lianyungang	LYG	24	34.82	119.51	2018
Nantong	NT	24	32.68	121.04	2017
Zhoushan	ZS	20	30.06	122.52	2015
Wenzhou	WZ	24	27.71	120.81	2016
Xiamen	XM	24	24.75	119.09	2017
Zhuhai	ZH	17	21.90	113.54	2016
